# Updates on the Causes, Diagnosis, and Management of Peritoneal Abscesses: A Systematic Review

**DOI:** 10.7759/cureus.48601

**Published:** 2023-11-10

**Authors:** Malik A Hussain, Riyad Y Al Laham, Hadeel T Alanazi, Taif A Alanazi, Reef A Alshammari, Basmah D Alrawaili

**Affiliations:** 1 Surgery, College of Medicine, Northern Border University, Arar, SAU; 2 Medicine, College of Medicine, Northern Border University, Arar, SAU

**Keywords:** abscess, peritoneal abscess, management, diagnosis, causes

## Abstract

Intra-abdominal complications such as peritoneal abscesses pose significant medical challenges. Over recent years, there has been a heightened focus on refining treatments for these conditions, such as optimal surgical techniques, drug therapies, and intervention methods. This paper aims to present a comprehensive overview of 10 research studies spanning various countries to highlight recent advancements and findings in the treatment and management of peritoneal abscesses. The paper reviewed 10 trials involving a total of 942 participants, covering diverse methodologies including randomized controlled trials, retrospective analyses, and phase 3 clinical trials. The research spanned countries such as the USA, Finland, Japan, Turkey, India, and China. Key findings included the notable benefits of laparoscopic interventions in appendiceal abscess treatments, which led to quicker recoveries and reduced readmissions compared to conservative approaches. Additionally, certain drug combinations, such as tazobactam/ceftolozane with metronidazole, showcased high clinical efficacy, particularly against resistant bacterial strains. Challenges persist in the early detection of intra-abdominal infections, emphasizing the pivotal role of antimicrobial treatments. Unique therapeutic approaches, like the use of strong acid-electrolyzed water (SAEW) in pediatric appendicitis cases, have proven effective in reducing surgical site infections. Intrabdominal complications such as peritoneal abscesses pose a real challenge. Early detection plays a critical role, which relies on using imaging techniques such as CT scans. Poorly managed mild intra-abdominal diseases can lead to the development of abscesses. Therefore, the implication of highly effective antibiotic combinations such as tazobactam/ceftolozane and metronidazole/ceftriaxone from the start can effectively combat challenging bacterial infections such as Gram-negative and anaerobic bacteria. Surgical procedures remain the most effective method to treat abscesses, and they are usually used as the last resort when drainage, laparoscopy, and other methods fail.

## Introduction and background

Peritoneal abscesses, localized collections of pus within the peritoneal cavity, arise from a variety of conditions and can be life-threatening if not promptly identified and treated [1]. A delay in diagnosis can result in severe complications, including sepsis, bowel obstruction, fistula formation, and even death [2]. As such, understanding the etiology, pathogenesis, and latest diagnostic and therapeutic modalities is of paramount importance for clinicians involved in the management of patients with suspected or confirmed peritoneal abscesses.

Historically, peritoneal abscesses have been attributed to various causes, including post-surgical complications, intra-abdominal infections like diverticulitis and appendicitis, and penetrating abdominal trauma [3]. In recent years, with the advent of advanced imaging techniques and more sophisticated laboratory diagnostics, our understanding of the causes and precipitating factors for peritoneal abscess formation has evolved [4].

The diagnostic strategies, too, have seen a shift over the past few decades. Previously, the diagnosis was primarily clinical, relying heavily on physical examination findings and patient history [5]. However, with the widespread availability and improved resolution of imaging modalities such as ultrasound, CT, and MRI, the accuracy and speed of diagnosis have substantially improved [6]. Notably, the role of laparoscopy in both the diagnostic and therapeutic realms has also expanded [7].

Management strategies for peritoneal abscesses have similarly undergone significant transformation. While surgical intervention was once the mainstay of treatment, the role of interventional radiology and percutaneous drainage, alongside targeted antibiotic therapy, has gained significant traction in recent years [8]. The push towards minimally invasive procedures and precision medicine requires clinicians to be updated on the latest evidence-based practices.

The diagnosis of a peritoneal abscess involves a multi-faceted approach that blends clinical evaluation with advanced imaging modalities. Historically, the diagnosis relied heavily on clinical signs and symptoms. However, with technological advancements, imaging techniques have become central to confirming the diagnosis and guiding treatment.

Typically, patients present with abdominal pain, tenderness, fever, and sometimes a palpable mass [9]. Elevated white blood cell counts and systemic signs of sepsis may be evident in lab tests [10]. Abdominal ultrasonography is a valuable initial tool in the diagnostic armamentarium. It's non-invasive, widely available, and can visualize fluid collections, particularly in the context of a suspected abscess. It helps in the identification of abscess location, size, and relation to adjacent structures and can guide percutaneous drainage if needed [11-13]. In situations where imaging is inconclusive and clinical suspicion remains high, diagnostic laparoscopy can be performed. This procedure allows direct visualization of the peritoneal cavity and can be therapeutic if an abscess is found and drained [14].

A multi-disciplinary approach, incorporating medical, radiological, and surgical strategies to optimize patient outcomes. Prompt and effective treatment is essential to prevent complications such as sepsis, bowel obstruction, and fistula formation [15, 16]. Under imaging guidance, usually ultrasound or CT, abscesses can be drained percutaneously. This minimally invasive approach has become a first-line treatment for many abscesses, especially when they are easily accessible and not complicated by fistulas or multi-loculated collections [17]. Some abscesses, especially those that are multi-loculated, associated with a fistula, or not amenable to percutaneous drainage (PD), require surgical intervention. The choice between open and laparoscopic surgery depends on the surgeon's experience, the patient's anatomy, and the specifics of the abscess [18-20]. The objective of this systematic review is to collate and present the latest evidence on the causes, diagnostic modalities, and management strategies for peritoneal abscesses, providing clinicians with an up-to-date resource to guide patient care.

## Review

Methodology

This was a systematic review conducted in September 2023. The Preferred Reporting Items for Systematic Reviews and Meta-Analyses (PRISMA) guidelines were followed for this systematic review.

Search strategy

To retrieve the relevant research, a thorough search was conducted across major databases, using PubMed primarily as a search engine for studies. We only searched in English. The following keywords were converted into PubMed Medical Subject Headings (MeSH) terms and used to find studies that were related: “causes,” "diagnosis," "management," and "peritoneal abscess". Boolean operators "OR" and "AND" matched the required keywords. Among the search results were publications in full English, freely available articles, and human trials.

The inclusion criteria considered studies that investigate the management of intra-abdominal abscesses such as peritoneal abscesses and liver abscesses, studies that employ preventive methods to avoid peritoneal abscesses, clinical trials, randomized controlled double-blinded trials, and freely accessible articles. Exclusion criteria included systemic reviews or meta-analyses, review articles, studies published 10 years ago or older, case reports, letters to the editors, and replies to conflicts.

Data extraction

Duplicates in the search strategy output were found using Rayyan (Qatar Computing Research Institute (QCRI), Doha, Qatar) [21]. To determine the titles’ and abstract relevance, the researchers used a set of inclusion and exclusion criteria to filter the combined search results. The reviewers carefully read each paper that met the requirements for inclusion. The authors provided other methods of resolving disputes with some thought. The authors extracted data about the study titles, authors, study year, country, participants, gender, diagnostic tool, main outcomes, and conclusion.

Strategy for data synthesis

Summary tables were created using information from pertinent research to give a qualitative overview of the results and study components. Following data extraction for the systematic review, the most effective strategy for utilizing data from the included study articles was selected.

Risk of bias assessment

Using the Risk of Bias in Non-randomized Studies of Interventions (ROBINS-I) risk of bias assessment approach for non-randomized trials of therapies, the included studies' quality was assessed [22]. The seven themes that were assessed were confounding, participant selection for the study, classification of interventions, deviations from intended interventions, missing data, assessment of outcomes, and choosing of the reported result.

Results

**Figure 1:**
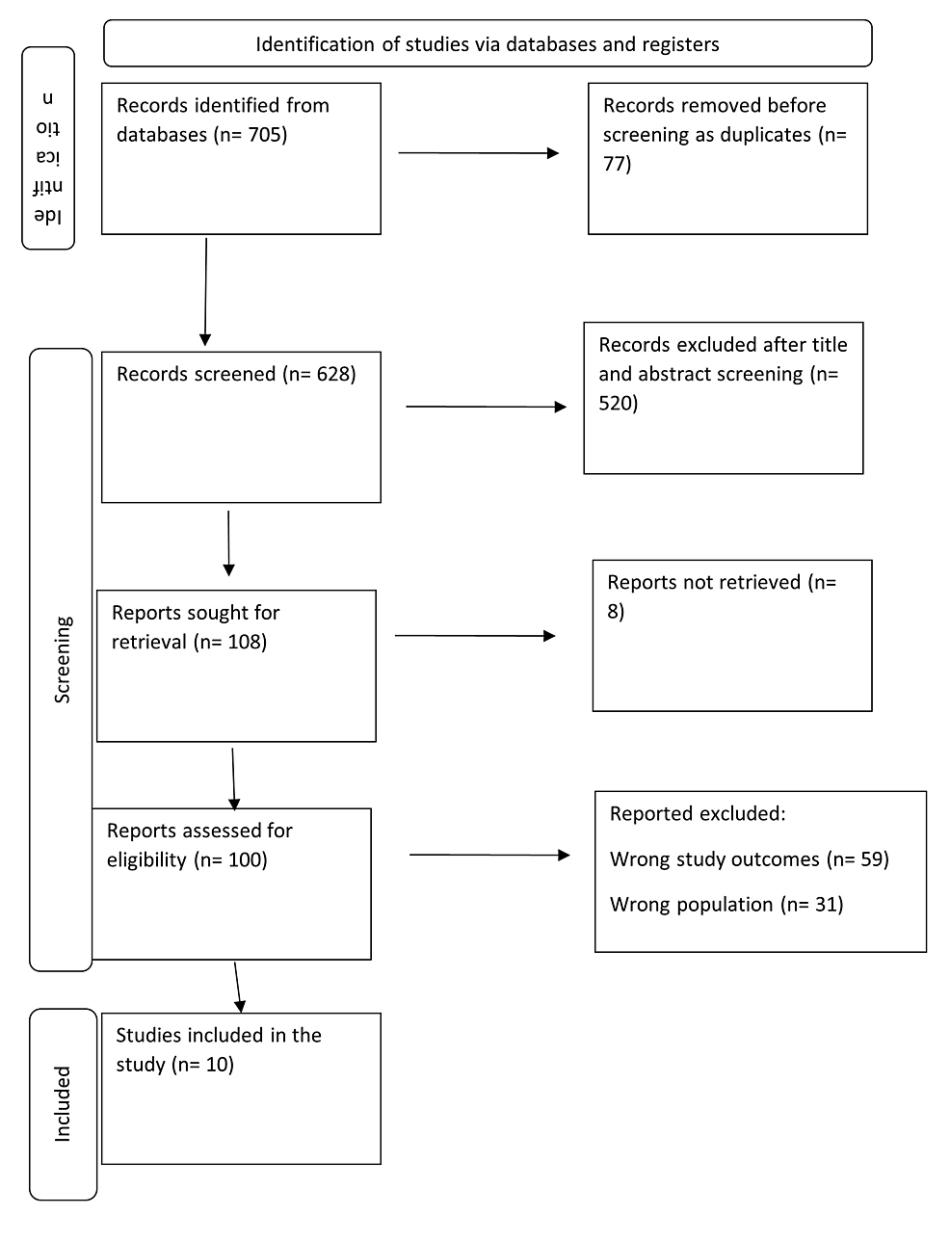
PRISMA flowchart summarizes the study selection process PRISMA: Preferred Reporting Items for Systematic Reviews and Meta-Analyses

A total of 705 study articles resulted from the systematic search, and 77 duplicates were deleted. Title and abstract screening were conducted on 628 studies, and 520 studies were excluded. One hundred and eight reports were sought for retrieval, and only eight articles were not retrieved. Finally, 100 studies were screened for full-text assessment; 59 were excluded for the wrong study outcomes, and 31 for the wrong population type. Ten eligible study articles were included in this systematic review. A summary of the study selection process is presented in Figure 1.

Table 1 displays the sociodemographic details and research methodologies from the provided research list.

**Table 1:** Sociodemographic details and research methodologies of the included studies

Study	Study design	Location	Participants	Age range (mean) in years	Males (%)
Dotson et al., 2015 [23]	Retrospective study	USA	30	15.4 ± 2.6	33%
Mentula et al., 2015 [24]	Randomized controlled trial	Finland	60	-	-
Mikamo et al., 2019. [25]	A multicenter, open-label, non-comparative study	Japan	100	47% were ≥65	67%
Anderson et al., 2020. [26]	Randomized controlled trial	USA	100 children	-	-
Kubota et al., 2015 [27]	Randomized controlled study	Japan	44	3-14	63.64%.
Akın et al., 2014. [28]	Prospective single-center study	Turkey	48	2-18 (11)	60%
Mikamo et al., 2015 [29]	Phase 3 clinical trial	Japan	38	-	-
Surya et al., 2020 [30]	Prospective, randomized study	India	100	22-74 (40)	88%
Sun et al., 2018 [31]	Prospective randomized trial	China	260	-	-
Lee et al., 2021 [32]	Prospective randomized trial	USA	162 children	-	-

The summary comprises 10 studies with an aggregate of 942 participants [23-32]. Regarding gender distribution, Dotson et al. [23] reported a male participation rate of 33%. Mikamo et al. [25] indicated a 67% male population, while Kubota et al. [27] specified 63.64%. Akın et al. [28] had a 60% male representation, and Surya et al. [30] featured the highest male percentage at 88%. However, some studies, such as Mentula et al. [24], Anderson et al. [26], Mikamo et al. [29], Sun et al. [31], and Lee et al. [32], did not specify the male percentage. Research was conducted across various countries, including the USA [23, 26, 32], Finland [24], Japan [25, 27, 29], Turkey [28], India [30], and China [31].

In terms of study design, most research employed randomized controlled trials or their variants [24, 26, 27, 30-32]. Specifically, Dotson et al. [23] used a retrospective approach, Mikamo et al. [25] adopted a multicenter, open-label, non-comparative study, Akın et al. [28] was a single-center prospective study, and Mikamo et al. [29] conducted a phase 3 clinical trial.

Age data varied among studies. Dotson et al. [23] had participants with a mean age of 15.4 ± 2.6 years, while Mikamo et al. [25] mentioned that 47% of the participants were aged 65 or older. Akın et al. [28] provided an age range of two to 18 years with an average age of 11 years, and Surya et al. [30] detailed an age range of 22-74 years with a mean age of 40 years. Kubota et al. [27] studied participants aged between three and 14 years. Other studies, namely Mentula et al. [24], Anderson et al. [26], Mikamo et al. [29], Sun et al. [31], and Lee et al. [32] did not mention specific age details.

Table 2 presents the clinical characteristics summary of the included studies.

**Table 2:** Clinical characteristics and outcomes of the included studies

Study	Results	Outcomes and conclusion
Dotson et al., 2015 [23]	Computed tomography (CT) was the primary method used for the initial diagnosis in 93% of the patients. Initial management: 18 patients received medical therapy, 10 had percutaneous drainage (PD), and two underwent surgery. Of the two who had surgery within one year, neither required further surgical procedures. Abscess management: There was a trend towards smaller abscesses being managed medically. Three patients from the medical group subsequently underwent PD. The duration of drain placement varied between PD and medical groups. Post-treatment course: Within a year post-hospitalization, there were no significant differences in readmissions, complications, or other metrics between the treatment groups. However, by the one-year mark, a significant number from both the medical and PD groups had undergone surgery: “64% of the total patients (18 out of 28)”.	Most patients with intra-abdominal abscess (IAA) require definitive surgical treatment, and there were no clear predictors of those who did not.
Mentula et al., 2015 [24]	The hospital stay was roughly similar between groups: four days for the laparoscopy group and five days for the conservative group. The laparoscopy group had a 10% risk for bowel resection and 13% for an incomplete appendectomy. Unplanned readmissions were notably lower in the laparoscopy group at 3%, compared to 27% in the conservative group. Additional interventions were needed for 7% of the laparoscopy group, mainly percutaneous drainage, whereas 30% of the conservative group required surgery, mainly due to recurrent abscesses or ineffective treatment. Open surgery occurred in 10% of the laparoscopy group and 13% of the conservative group. The laparoscopy group had a 90% uneventful recovery rate, significantly higher than the 50% in the conservative group.	When performed by experienced professionals, laparoscopic surgery is a safe and viable primary treatment option for appendiceal abscesses. It offers the benefits of fewer readmissions and fewer additional interventions compared to conservative treatment, while the duration of hospital stay remains comparable.
Mikamo et al., 2019. [25]	Out of the 100 participants, 92 were evaluated for clinical efficacy. Clinical response rates were high, with cholecystitis at 92.3%, liver abscess at 100%, intra-abdominal abscess at 93.5%, and peritonitis at 90.2%. The per-subject microbiological response rate was 90.2%. Major infections included the appendix (53 participants) and gallbladder (30 participants), with 68% having an IAA and 67% having peritonitis; 44% underwent appendectomy, and 24% had cholecystectomy. Baseline antibacterial therapy failure was observed in 16% of subjects.	Tazobactam/ceftolozane, combined with metronidazole, showcases a promising clinical response rate for various intra-abdominal infections, especially in the elderly and those with renal impairment. It also demonstrated effectiveness against extended-spectrum beta-lactamase (ESBL)-positive *Enterobacteriaceae*.
Anderson et al., 2020. [26]	Both treatment groups had similar baseline characteristics. In the povidone-iodine (PVI) group, 12% of patients developed postoperative IAA, compared to 16% in the no irrigation (NI) group. Bayesian analysis showed an 89% likelihood that PVI reduces IAA. Secondary results indicated probable benefits for the PVI group, including fewer ED visits, reduced readmissions, and shorter hospital stays. Notably, there was a 96% probability of a reduced 30-day length of stay (LOS) for PVI patients, which was statistically significant.	The study suggests that PVI irrigation for children with perforated appendicitis might reduce the incidence of postoperative IAA and decrease the length of hospital stay.
Kubota al., 2015 [27]	Strong acid-electrolyzed water (SAEW) showed no adverse effects in Group E. Both groups had similar results in the bacterial evanescence ratio of ascitic fluid and the bacterial count reduction post-peritoneal lavage. However, Group E demonstrated a significantly lower incidence of surgical site infections (SSI) at 0% compared to Group S's 20%. Despite this, there was no difference in the duration of fever, positive C-reactive protein, leukocytosis, or the length of hospital stays between the groups. Additionally, the infections in Group S were diverse, with multiple bacterial species, while those in Group E were mostly limited to *Escherichia coli (E. coli)*.	Using SAEW for peritoneal lavage and wound cleaning did not result in any adverse effects and proved effective in preventing SSIs. The study suggests that SAEW could be a safer and more effective alternative to saline for treating perforated appendicitis in children.
Akın M et al., 2014. [28]	Pre-appendectomy aerobic cultures revealed microorganisms in 38% of patients, with* E. coli* dominating. Post-appendectomy aerobic cultures showed bacteria in 14.6% of patients, indicating a significant reduction. Anaerobic microorganisms, with *Bacteroides fragilis* being predominant, were present in 25% of pre-appendectomy samples and 8.3% post-appendectomy, showing a significant bacterial count decrease.	The research indicates that undergoing a laparoscopic appendectomy doesn't raise the risk of infections inside the abdomen, especially from anaerobic bacteria. When comparing bacterial counts before and after the surgery, there was a notable drop in the numbers.
Mikamo et al., 2015 [29]	The combined metronidazole (MNZ) and ceftriaxone (CTRX) treatment exhibited high clinical efficacy. For those with infectious peritonitis or abdominal abscess, the clinical efficacy rate was 100% (20 out of 20 subjects). In subjects with pelvic inflammatory disease (PID), the efficacy rate stood at 90% (nine out of 10 subjects). The bacterial eradication rates were 100% in both categories. A noteworthy outcome was the treatment's effectiveness against a specific resistant *Bacteroides fragilis* strain. The most commonly reported side effect was diarrhea (23.7%), followed by nausea (5.3%).	The intravenous combination of MNZ and CTRX demonstrated high efficacy and was well-received by Japanese patients with infectious peritonitis, abdominal abscess, or PIDs. The treatment showed promise for tackling anaerobic infections. There were some side effects, but no new safety concerns arose. This treatment could be a valuable tool for addressing anaerobic infections.
Surya et al., 2020 [30]	Both percutaneous needle aspiration (PNA) and percutaneous catheter drainage (PCD) had high success rates (88% and 92%, respectively), with no significant statistical difference between them. However, the average hospital stay and time between intervention and discharge were shorter in the PNA group, being 6.8 days and 5.9 days, compared to the PCD group's 10.5 days and 10.2 days. A significant complication noted was peritonitis due to a peri-catheter leak in the PCD group.	Both PNA and PCD are effective in treating liver abscesses. However, due to the shorter hospital stay, patient safety, convenience, simplicity of the procedure, and cost-efficiency, needle aspiration is recommended as the primary treatment for liver abscesses larger than 5 cm. Continuous catheter drainage should be considered in cases where a second needle aspiration attempt fails.
Sun et al., 2018 [31]	From January 2015 to June 2016, 260 patients were studied. The irrigation technique led to extended operation times. No notable difference was observed in wound infection rates between groups. However, the irrigation group showed a reduced rate of postoperative intra-abdominal abscess, faster anal exsufflation, shorter hospital stays, and decreased hospital costs compared to the suction-only group.	Ample irrigation of the peritoneal cavity during laparoscopic appendectomy may reduce the risk of postoperative intra-abdominal abscess in adults with severe appendicitis. Additionally, these patients experienced quicker postoperative recovery and had lower hospital expenses.
Lee et al., 2021 [32]	Among 162 patients, no noticeable variations in age, weight, or duration of symptoms emerged. Moreover, length of hospital stay, treatment durations, and antibiotic-related complications remained consistent across groups. However, the piperacillin-tazobactam (PT) group experienced significantly reduced IAA rates, fewer CT scans, and fewer ER visits compared to the CM group. The most significant factor leading to IAA was the use of 2-drug therapy (ceftriaxone and metronidazole (CM)) over PT.	For children with perforated appendicitis, postoperative treatment using PT is more effective than the standard two-drug therapy (CM). There's no observed increase in antibiotic-related complications or the length of antibiotic exposure with PT.

In a retrospective study by Dotson et al. (2015) [23], pediatric Crohn’s disease patients with intra-abdominal abscesses were examined. Computed tomography scans were the primary diagnostic tool in 93% of these cases. When initial treatments were analyzed, it was clear that most patients, around 64% by the one-year mark, ultimately required surgical treatment, even though various modalities like medical therapy, PD, and surgery were initially applied. There were no discernible predictors to determine which patients wouldn't need surgery. Mentula et al. (2015) [24] explored immediate laparoscopic surgery for appendiceal abscess patients, comparing it with conservative treatment. The study revealed that laparoscopic surgery performed by experts presented fewer readmissions, fewer additional interventions, and comparable hospital stay durations, making it a viable primary treatment choice.

Mikamo et al. conducted two separate studies in 2019 and 2015. [25,29] The 2019 study demonstrated the efficacy of a combined tazobactam/ceftolozane with metronidazole regimen in treating Japanese patients with intra-abdominal infections. This combination displayed impressive clinical response rates and was particularly effective against extended-spectrum beta-lactamase (ESBL)-positive *Enterobacteriaceae*. The 2015 study evaluated the combination of intravenous metronidazole and ceftriaxone for specific intra-abdominal infections. The results were promising, revealing high efficacy and good tolerance, especially against resistant anaerobic infections.

Anderson et al. (2020) [26] focused on pediatric patients with perforated appendicitis. They aimed to discern if povidone-iodine (PVI) irrigation could reduce postoperative intra-abdominal abscess (IAA) occurrence. The study found that PVI potentially minimized the postoperative IAA risk and shortened the hospital stay. Kubota et al. (2015) [27] assessed the efficacy of strong acid-electrolyzed water (SAEW) in treating perforated appendicitis in children. Their findings suggest that SAEW, when used for peritoneal lavage and wound cleaning, efficiently prevents surgical site infections and can be a potential alternative to saline.

Akın et al. (2014) [28] investigated the role of laparoscopic appendectomy concerning postoperative intra-abdominal infections. They found a significant reduction in bacterial counts after the procedure, indicating that the risk of postoperative infections isn't heightened by the laparoscopic approach. Surya et al. (2020) [30] studied the effectiveness of two treatments for liver abscesses larger than 5 cm: intermittent percutaneous needle aspiration (PNA) and continuous percutaneous catheter drainage (PCD). Both treatments were effective; however, needle aspiration stood out due to its numerous benefits, including a shorter hospital stay.

Sun et al. (2018) [31] looked into the benefits of extensive peritoneal irrigation during laparoscopic appendectomy for severe appendicitis. They found that ample irrigation could potentially reduce postoperative intra-abdominal abscess risk, shorten recovery time, and decrease hospital costs. Lastly, Lee J et al. (2021) [32] compared postoperative monotherapy piperacillin and tazobactam (PT) with standard two-drug therapy (ceftriaxone and metronidazole (CM)) for children with perforated appendicitis. The findings suggested that PT is more effective than CM in reducing intra-abdominal abscess occurrences without any increase in complications. Together, these studies offer comprehensive insights into the diagnosis and management strategies for intrabdominal or peritoneal abscesses.

Discussion

This paper provides a comprehensive overview of 10 research studies involving 942 participants. The studies reveal varying gender distribution rates, with male participation ranging from 33% to 88% across different research papers, although a few studies did not specify gender ratios. Geographically, the research spans several countries, including the USA, Finland, Japan, Turkey, India, and China. The methodologies adopted primarily include randomized controlled trials, with a few studies employing retrospective, multicenter, single-center prospective, and phase 3 clinical trial approaches. Age data also displayed variations, with some studies focusing on specific age groups while others lacked detailed age information.

In recent years, a multitude of studies have examined treatment methodologies for various intra-abdominal conditions, focusing primarily on optimal surgical techniques, interventions, and drug therapies. A retrospective examination of pediatric Crohn’s disease patients by Dotson et al. (2015) [23] identified a significant number of children developing intra-abdominal abscesses between 2000 and 2012. The study emphasized that the majority of patients diagnosed through CT scans subsequently needed surgical treatments. Interestingly, there weren't any discernible predictors that could determine which patients would not require surgical intervention.

For a patient who is hemodynamically stable but showing signs of localized peritonitis, it's crucial to use imaging to determine the extent of contamination and how well it's been contained. If there's a contained perforation without broad contamination of the abdomen, it might be treated with just antibiotics and possibly percutaneous drainage for larger abscesses. One application of this is in determining the severity of perforated diverticulitis. Computed tomography scans have been used to classify diverticulitis severity, and while they've been largely accurate in staging the majority of patients, there have been instances where patients with specific types of peritonitis were misidentified. This distinction is important, especially as researchers are now examining the potential role of laparoscopic lavage in certain stages of diverticulitis. [33-36]

Similarly, Mentula et al. (2015) [24] directed attention toward appendiceal abscess patients, assessing whether immediate laparoscopic surgery could lead to quicker recoveries than conservative treatments. Their study, which comprised 60 adult patients, found that the laparoscopy group had fewer readmissions and needed fewer additional interventions than their conservative counterparts. This suggests that experienced surgeons can leverage laparoscopy as a primary treatment method with notable benefits.

Draining peritoneal abscesses with a needle can offer several advantages, such as providing immediate relief from symptoms, reducing the risk of complications, and allowing for a less invasive procedure compared to surgery. However, it is important to consider the potential disadvantages, including the risk of infection, incomplete drainage, and the possibility of causing damage to surrounding tissues. It is crucial for healthcare professionals to carefully weigh the benefits and risks of this procedure and to ensure that it is performed with the utmost care and consideration for the patient's well-being [37].

Focusing on drug therapies, Mikamo et al. (2019) [25] evaluated the efficacy of tazobactam/ceftolozane in tandem with metronidazole in treating Japanese patients with complicated intra-abdominal infections. Their results painted an encouraging picture, with high clinical response rates observed across multiple conditions. Additionally, the drug combination demonstrated efficacy against ESBL-positive *Enterobacteriaceae*. Anderson et al. (2020) [26] explored an alternative approach to reducing postoperative intra-abdominal abscesses in children with perforated appendicitis. Comparing PVI irrigation with no irrigation, their Bayesian analysis posited a high likelihood that PVI is more beneficial, as indicated by reduced ED visits, readmissions, and overall hospital stays.

One of the primary challenges with complex intra-abdominal infections is their early detection. While there are standardized antimicrobial treatments available that have shown effectiveness, either as standalone or combination therapies, the routine use of treatments against enterococci isn't always advised. However, it can be beneficial under specific circumstances, such as septic shock in patients with prior extended cephalosporin treatments, those with compromised immune systems at risk for blood infections, individuals with artificial heart valves, or repeated severe infections. For patients with extended hospitalization and antibacterial treatment, the risk of resistant pathogens is higher. If there's evidence of *Candida *involvement or patients at risk of invasive candidiasis, antimicrobial coverage is advised. Typically, the antimicrobial treatment should last five to seven days, but if sepsis persists after a week, further diagnostics and possibly surgical intervention might be needed [37, 38].

Kubota et al. (2015) [27] undertook an innovative study based on prior experiments on rats. They examined the effectiveness of SAEW in treating perforated appendicitis in children. Their findings indicate that SAEW when used for peritoneal lavage and wound cleaning, can effectively reduce surgical site infections without adverse effects. Akın et al. (2014) [28] aimed to understand the role of laparoscopic appendectomy in postoperative intra-abdominal infections. Their microbial analysis revealed a significant reduction in bacterial counts post-surgery, reaffirming the safety of laparoscopic appendectomy in such scenarios.

*Bacteroides fragilis* is an obligate anaerobic and Gram-negative bacillus. The combination of ceftriaxone and metronidazole is suitable for the treatment of infections caused by *Bacteroides fragilis*. A study by Mikamo et al. (2015) [29] sought regulatory approval for the combined use of metronidazole and ceftriaxone for patients in Japan with specific intra-abdominal conditions. Their treatment presented high efficacy, especially against anaerobic infections like a resistant *Bacteroides fragilis* strain. Liver abscesses were the focus of Surya et al. (2020) [30], who compared PNA with PCD for treatment. Their conclusion recommended PNA due to its various benefits, including a shorter hospital stay and overall patient safety.

Sun et al. (2018) [31] revisited the domain of laparoscopic appendectomy but added a new dimension: extensive peritoneal cavity irrigation. Their findings suggest that this approach can be beneficial for adult patients with severe appendicitis, reducing postoperative complications and expediting recovery. Lastly, Lee et al. (2021) [32] delved into postoperative therapies for children diagnosed with perforated appendicitis. By comparing postoperative monotherapy PT with standard two-drug therapy (CM), their results favored PT, showcasing its efficacy in reducing intraabdominal abscesses without increasing complications.

Postoperative care for peritonitis patients is a critical and complex process that requires a high level of attention and expertise. Due to the severity of their condition and the potential for complications, it is essential to provide rigorous care to ensure the best possible outcomes for these patients. One of the key components of postoperative care for peritonitis patients is aggressive intravenous fluid therapy. This is particularly important for patients who are experiencing ongoing fluid loss from the inflamed peritoneal cavity. By providing a steady supply of fluids, healthcare professionals can help stabilize the patient's condition and prevent further complications [37]. In addition to fluid therapy, regular assessment and correction of electrolytes and acid-base balance are essential. Imbalances in these areas can have serious consequences for peritonitis patients, so it is crucial to monitor and address any issues that arise. Enteral feeding is preferred for peritonitis patients, but it may be challenging for anorectic patients unless GI feeding tubes are placed during surgery. This presents a unique challenge for healthcare professionals, who must find ways to ensure that patients receive the nutrition they need to support their recovery. Finally, postoperative hypotension should be managed with vasopressors, but only after addressing underlying hypovolemia. This requires a careful and thoughtful approach, as the use of vasopressors can have significant implications for the patient's overall condition [37, 39].

## Conclusions

A peritoneal abscess is a complex medical condition that requires a thorough understanding of its causes, an accurate diagnosis, and prompt and appropriate management. By utilizing a multidisciplinary approach and individualized treatment plans, healthcare professionals can effectively manage peritoneal abscesses and improve patient outcomes. Further research and advancements in medical technology will continue to enhance our understanding and management of this challenging condition. Across all the included studies, a common thread emerges in the continual search for effective, safe, and patient-centric interventions for intra-abdominal sepsis. The medical community's commitment to refining techniques, testing new drug combinations, and leveraging advanced surgical approaches is evident. As these studies demonstrate, such commitment invariably leads to advancements that better the lives of patients worldwide.
